# Migrant-Local Differences in the Relationship Between Oral Health, Social Support, and Loneliness Among Older Adults in Weifang, China: Cross-Sectional Study

**DOI:** 10.2196/66061

**Published:** 2025-03-13

**Authors:** Hui Liu, Jieru Wang, Rui Chen, Xixing Xu, Mingli Pang, Kaiyuan Feng, Bingsong Li, Qinling Li, Ziwei Qin, Shuyi Yan, Nabila Ibn Ziyat, Fanlei Kong

**Affiliations:** 1 Department of Social Medicine and Health Management, School of Public Health, Cheeloo College of Medicine, Shandong University, 44 Wenhuaxi Road, Jinan, China, 86 19966534511; 2 NHC Key Lab of Health Economics and Policy Research, Shandong University, Jinan, China; 3 Center for Health Management and Policy Research, Shandong University (Shandong Provincial Key New Think Tank), Jinan, China; 4 Editorial Office of Chinese Rural Health Service Administration of Publishing Center, Anhui Medical University, Hefei, China

**Keywords:** loneliness, oral health, social support, migrant older adults, local older adults

## Abstract

**Background:**

Increased aging and accelerated urbanization have led to the migration of older adults within China. Migrant older adults (MOAs) may experience physical and psychological discomfort in influx cities, and they are a vulnerable group that has emerged in the course of fast urbanization. Previous studies have confirmed the association between oral health and loneliness as well as the relationship between social support and loneliness; however, no research has been done to clarify the underlying mechanisms and the migrant-local difference between oral health, social support, and loneliness.

**Objective:**

This study aimed to test the association between oral health, social support, and loneliness among Chinese older adults, as well as the migrant-local difference on the above relationship.

**Methods:**

Multistage cluster random sampling was used to enroll a total of 1205 participants, including 613 MOAs and 592 local older adults (LOAs). Loneliness was assessed by the 6-item short-form UCLA Loneliness Scale; oral health was measured via the Chinese version Geriatric Oral Health Assessment Index (GOHAI); social support was evaluated by the Social Support Rating Scale (SSRS). Descriptive analysis, *χ*^*2*^ tests, and *t* tests were conducted. Multigroup structural equation modeling (SEM) was employed to clarify the migrant-local difference on the association between oral health, social support, and loneliness among MOAs and LOAs.

**Results:**

The mean score of loneliness was 8.58 (SD 3.032) for MOAs and 8.00 (SD 2.790) for LOAs. Oral health and social support were found to be negatively related to loneliness among MOAs and LOAs; the standardized direct effects for MOAs were −0.168 and −0.444 (*P*<.001), and they were −0.243 and −0.392 (*P*<.001) for LOAs, respectively. Oral health generated a direct positive effect on social support, and the direct effect was 0.186 for MOAs (*P*<.001) and 0.247 for LOAs (*P*<.001).

**Conclusions:**

Loneliness was fairly low among older adults in Weifang, China, while MOAs showed higher loneliness than LOAs. Oral health had both direct and indirect negative effects on loneliness among MOAs and LOAs, with no significant path differences between MOAs and LOAs. Social support was found to be negatively associated with loneliness for both MOA and LOA, while the association was stronger among MOAs than LOAs. Oral health exerted a significantly positive effect on social support for both MOAs and LOAs, while no significant difference existed between them. Measures should be taken by the government, society, and families to increase social support, improve oral health, and further reduce loneliness among MOAs and LOAs.

## Introduction

China has one of the fastest growing aging populations in the world [1]. According to the data of China’s Seventh National Census, the number of people aged 60 years or above was 264.02 million, accounting for 18.70% (the number of people aged 65 years or above was 190.64 million, accounting for 13.50%). Compared with 2010, the proportion of people aged 60 years or above increased by 5.44% [2]. As the population ages, concern regarding older adults’ health status has also increased.

In the past few decades, China has experienced a rapid increase in economic level and an acceleration of urbanization, which also caused an economic gap across different cities and may further lead to population migration. Due to the traditional Chinese culture, which highlights the family union, more and more older adults move to live with their adult children. Existing studies refer to the older adults who leave their hometown to migrate with their children as migrant older adults (MOAs) [3]. Previous studies found that MOAs had difficulties in developing social networks and social integration, which would further affect their mental health [4,5], life satisfaction [6], and quality of life [7]. Based on “adaptive theory” [8] and “social support buffering model” [9], MOAs face increased adaptation stress and social isolation, which may lead to higher reliance on social support networks to cope with the challenges posed by migration. Thus, it is also important to pay more attention to the health status of MOAs, in addition to that of local older adults (LOAs).

Loneliness is defined by Weiss [10] as a subjective feeling formed when individuals perceive that they lack satisfying interpersonal relationships and there is a gap between their desire to connect and the actual situation of connection. Donovan et al [11] found that 17.6% of US older adults felt lonely much of the time during the preceding week. Guo et al’s [12] nationwide study found that 24.3% of older people reported they were lonely in China. Another study found that nearly one-fourth of Chinese older adult participants felt lonely [13]. In terms of MOAs, one longitudinal study showed that migrants from non–English-speaking countries reported higher levels of loneliness, as compared with the native-born, non-Indigenous Australians [14]. Some studies indicated that loneliness was associated with some negative health outcomes [15,16], such as poor physical health [17] and poor mental health [18,19]. Moreover, another national study in China clarified that a high degree of loneliness could further reduce the life satisfaction of older adults [20]. A cohort study from northern and southern Europe showed that loneliness was associated with a decreased quality of life among older adults [21]. Therefore, it is important to pay more attention to older adults’ loneliness and find possible ways to reduce their loneliness.

Oral health has been defined by the World Health Organization (WHO) as a state of being free from chronic mouth and facial pain, oral and throat cancer, oral infection and sores, periodontal (gum) disease, tooth decay, tooth loss, and other diseases and disorders that limit an individual’s capacity in biting, chewing, smiling, speaking, and psychosocial well-being [22]. The Fourth Chinese Oral Health Epidemiological Survey Report demonstrated that the prevalence of dental caries in older adults’ permanent teeth was fairly high, implying serious oral health conditions [23]. Oral health, as a part of general health, is an important determinant of loneliness. One study examined the relationship between oral health and mental health issues and found that oral health was negatively associated with depression [24]. However, few studies have examined the association between oral health and loneliness. A study among English older adults demonstrated that oral health–related quality of life was identified as an independent risk factor for loneliness both cross-sectionally and longitudinally, and older adults with oral impacts had a significantly higher risk of being lonely than their counterparts without any oral impacts [25]. A previous study also found that the fewer number of teeth, the more likely both Japanese and British older adults were to report social isolation or loneliness [26]. Studies also clarified that the degenerative changes of oral function could lead to eating difficulties and imbalanced nutritional intake, which in turn affects physical and mental health and may increase the loneliness of older adults [27,28]. Thus, it is important to test the empirical association between oral health and loneliness among older adults.

Social support refers to the material and moral help provided by various parties in society, including family, relatives, friends, colleagues, organizations, and trade unions [29]. A meta-analysis has pointed out that increased social support would be supportive of successful aging among older adults [30]. Existing research also found that social support could not only provide the necessary resources (such as financial help and emotional help) for older adults to cope with challenges, but also had a huge positive impact on their physical and mental health [30,31]. Conversely, previous studies clarified that a lack of social support would cause adverse outcomes, such as limitations on activities of daily living [32] and poor quality of life [33]. The association between social support and loneliness has been widely explored. A previous study pointed out that social support was negatively associated with loneliness among older adults [34]. Chung and Kim [35] found that social support was critical in lowering loneliness in middle-aged and older adults. Studies from different countries indicated that the changes in older people’s social networks and social support resulted in a significant onset of loneliness during the COVID-19 pandemic [36,37].

A previous study indicated that social support was related to older adults’ oral health [38]. A nationwide study in Britain demonstrated social support was associated with the oral health status and oral health behavior of older people [39]. A study conducted in Germany clarified that impaired oral health–related quality of life was positively correlated with lower social support among older seniors [40]. A study had shown that oral condition was closely related to facial appearance, which can affect social image and confidence and negatively affect relationships [41]. A cross-national comparative study found that oral health was strongly associated with social isolation, with poorer oral health accelerating social isolation and thus reducing individuals’ perceived social support [26]. Therefore, oral health has a critical impact on social support in older adults, and it is essential to study the effects of both in MOAs and LOAs.

Based on the above literature review, no study was found that determined the association between oral health, social support, and loneliness, and no study has ever compared the difference between MOAs and LOAs regarding the above relationship. Thus, this study aimed to (1) clarify the association between oral health, social support, and loneliness and (2) test whether a statistically significant difference exists for the above relationship.

## Methods

### Sample Collection

The data were collected in Weifang City, Shandong Province, China in August 2021. The gross domestic product of Weifang City was 701.06 billion Chinese yuan in 2021 (US $96.9 billion at 2025 conversion rate) [42]. Weifang governed 10 districts and 2 counties (59 subdistricts and 59 towns) until July 2020 [43]. The total population of Weifang City was 9.3 million by the end of 2020 according to the Seventh National Census. In 2020, nearly 2.38 million of its whole population constituted migrants from other counties, cities, or provinces, with a variety of sociodemographic and cultural backgrounds [44]. Thus, two groups of older adults were recruited in this study. For MOAs, the inclusion criteria were: (1) aged ≥60 years; (2) their Hukou is not in the present place (Hukou is one of China’s oldest tools for population control; it is essentially a household registration permit, similar to an internal passport, which defines where people are registered, not where they currently reside [45]); and (3) an ability and willingness to communicate with surveyors. For LOAs, the inclusion criteria were (1) and (3) from the previous list.

Multistage cluster random sampling was conducted to select the samples of MOAs and LOAs. In the first stage, 4 of 12 districts were selected as the primary sampling units (PSUs), considering the economic development and geographic location. In the second stage, 1 subdistrict was selected from each district (PSU), and a total of 4 subdistricts were taken as the secondary sampling units (SSUs). In the last stage, 4 communities were chosen from the SSUs as the tertiary sampling units (TSUs); that is, 1 community was selected from each of the subdistricts chosen previously. All the MOAs as well as the LOAs who met the above criteria constituted the total study sample. Initially, 616 MOAs and 592 LOAs were selected for interviews. However, 3 MOAs were excluded for answering the questionnaire incorrectly or incompletely. Ultimately, 613 MOAs and 592 LOAs were included in the database. The detailed sample selection process is shown in Multimedia Appendix 1.

### Measurement

#### Sociodemographic Characteristics

The section on sociodemographic characteristic traits included the following: gender (man, woman); age group (60‐64, 65‐69, 70‐74, 75‐79, ≥80 years); Hukou (rural, urban); marital status (married, single); education level (illiterate, primary school, junior high school, high school and above); pension (Yes, No).

#### Oral Health

The Chinese version of the Geriatric Oral Assessment Index (GOHAI) was used to measure participants’ oral health status. This tool is primarily used to assess the self-reported oral health of older adults and is widely used in China [46]. The Chinese version of the GOHAI is divided into 12 items and 3 subdimensions designed to assess different aspects of oral health: (1) physical functioning (four items), (2) psychosocial functioning (five items), and (3) pain or discomfort (three items). GOHAI scores could be divided into the following 3 categories: (1) 50 and below are defined as low oral health, (2) 51‐56 as fair-to-moderate oral health, and (3) 57‐60 as high oral health. In the previous study, the GOHAI scores also had good reliability and validity [47]. In this study, the Cronbach α coefficient was 0.853, indicating that the scale had good reliability.

#### Social Support

The Social Support Rating Scale (SSRS) was used to measure the social support of MOAs and LOAs, including 10 types of support: friends, residents, neighbors, colleagues, family members, financial, comfort, conversation, help, and activities [48]. The social support scale has 10 items, including 3 dimensions of objective support (3 items), subjective support (4 items), and utilization of social support (3 items). The range of the total score of the scale is 12‐66. A higher total social support score means the subject received more social support. The SSRS has been proven to have good reliability and validity in practice and has been widely used in China [49,50]. The Cronbach α coefficient was 0.822, implying this scale had good reliability.

#### Loneliness

The 6-item version of the UCLA Loneliness Scale (ULS-6) was used to assess the loneliness of participants, which excluded 2 reverse-scored items from the 8-item UCLA Loneliness Scale. The scale measures loneliness caused by the discrepancy between the level of desired social engagement and that which is actually experienced. The options are on a Likert scale: l=never, 2=rarely, 3=sometimes, and 4=always, with a total score ranging from 6‐24. Previous studies have demonstrated good reliability and validity in a Chinese population [51,52]. In this study, the Cronbach α coefficient of the ULS-6 was 0.82, indicating the scale had good reliability.

### Statistical Analysis

This study used SPSS (version 24.0; IBM Corp) and AMOS (version 24.0; IBM Corp) to perform the data analysis.

The samples’ sociodemographic characteristics in this study were characterized by using descriptive statistics, including frequency (%) and mean (SD). The difference between sociodemographic characteristics of MOAs and LOAs was determined using the *χ*^*2*^ test, while the *t* test was used to explore the difference in oral health (3 dimensions), life satisfaction (5 items), and loneliness (6 questions) of MOAs and LOAs. *P* values less than .05 were considered statistically significant. The above analyses were performed using SPSS (version 24.0; IBM Corp).

A hypothesized structural equation model was set to analyze the relationship between oral health, social support, and loneliness among MOAs and LOAs in Shandong Province. The maximum likelihood estimation method was used to evaluate the hypothesized model’s fit. The structural equation modeling (SEM) process model consisted of 2 categories of variables: latent variables and observed variables. The latent variables were oral health, life satisfaction, and loneliness. The 3 observed variables of oral health included physical functioning, psychosocial functioning, and pain and discomfort. The 3 observed variables of social support were objective support, subjective support, and utilization of social support. The 6 observed variables of loneliness were the 6 items of the ULS-6. All SEM analyses were performed using AMOS (version 24.0; IBM Corp).

The following model fitness indexes were used to judge the fit of the hypothesized model: CMIN (Chi-square value, *χ*^*2*^), degrees of freedom (*df*), *P* value of the *χ*^*2*^ test, root mean square error of approximation (RMSEA), the goodness-of-fit index (GFI), the adjusted goodness-of-fit index (AGFI), and the comparative fit index (CFI). In this study, the models would be regarded to be well-fitted when *P*>.05, GFI>0.90, AGFI>0.90, and RMSEA<0.05 [53]. *P* value is easily influenced by sample size under many conditions, so it was only demonstrated in this study and not used as a criterion for judgment [54].

In the multigroup analysis, various parameters are restricted to find the most suitable path model. Five models were displayed in this study, namely M1 (MOA model), M2 (LOA model), M3 (unconstrained model), M4 (measurement weights model), and M5 (structural weights model). M1 and M2 were models fitted based on the sample data of two groups, and M3-M5 were obtained by adding conditions gradually restricted from the initial unconstrained model [55]. The multigroup model invariance was determined before the discussion of the MOA and LOA difference in the structural model of SEM. The change in CFI (∆CFI) and the change in RMSEA (∆RMSEA) were used to assess the measurement invariance between unconstrained and constrained multigroup analyses [55]. ∆CFI is independent of both model complexity and sample size, as well as the overall fit measurements. ∆CFI<0.010 indicates that we obtained measurement invariance across groups [56]. For the ∆RMSEA, with more than 300 samples, ∆RMSEA less than 0.015 implies that measurement invariance has been successfully established [57]. After the multigroup model invariance test was passed, we determined whether there were path differences between the different groups based on the model outputs, and paths with an absolute value of the critical ratio greater than 1.96 indicate a significant difference in the coefficients between the two groups (*P*<.05) [58].

### Ethical Considerations

The research program was reviewed and approved by the ethical committee of Shandong University (number 20180225). For the original data collected, all participants had given informed consent to our study and were well aware of their right to withdraw from the study at any time. Our data have been completely anonymized and there is no information to identify the participants.

## Results

### Sample Characteristics

Table 1 shows the demographic characteristics of the participants, with a total of 1205 older adults included in the data analysis, of which 613 were MOAs and 592 were LOAs. Overall, 885 (73.4%) of the total sample were women while 320 (26.6%) were men; more than half (64.7%) of participants belonged to the 60‐69 year old group; nearly half of older adults had a rural Hukou; 971 of 1205 participants (80.6%) were married; approximately four-fifths of older adults (n=975, 80.6%) were educated; and 785 (65.1%) older adults have a pension.

**Table 1.** Characteristics of participants and disparity between MOAs^a^ and LOAs^b^.

Variables		Total (n=1205), n (%)	MOA (n=613), n (%)	LOA (n=592), n (%)	Chi-square (*df*)	*P* value
**Gender**					0.083 (1)	.79
	Men	320 (26.6)	165 (26.9)	155 (26.1)		
	Women	885 (73.4)	448 (73.1)	437 (73.8)		
**Age group (years**)					139.631 (4)	<.001
	60‐64	436 (36.2)	271 (44.2)	165 (27.9)		
	65‐69	344 (28.5)	215 (35.1)	129 (21.8)		
	70‐74	175 (14.5)	76 (12.4)	99 (16.7)		
	75‐79	105 (8.7)	27 (4.4)	78 (13.2)		
	≥80	145 (12)	24 (3.9)	121 (20.4)		
**Hukou**					507.268 (1)	<.001
	Rural	649 (53.9)	525 (85.6)	124 (20.9)		
	Urban	556 (46.1)	88 (14.4)	468 (79.1)		
**Marital status**					43.045 (1)	<.001
	Married	971 (80.6)	539 (87.9)	432 (73)		
	Single	234 (19.4)	74 (12.1)	160 (27)		
**Education level**					41.675 (3)	<.001
	Illiterate	230 (19.1)	161 (26.2)	69 (11.7)		
	Primary school	403 (33.4)	185 (30.2)	218 (36.8)		
	Junior high school	338 (28)	158 (25.8)	180 (30.4)		
	High school and above	234 (19.4)	109 (17.8)	125 (21.1)		
**Pension**					106.504 (1)	<.001
	Yes	785 (65.1)	314 (51.2)	471 (79.6)		
	No	420 (34.9)	299 (48.8)	121 (20.4)		

a MOAs: migrant older adults.

b LOAs: local older adults.

The disparity between MOAs and LOAs was statically significant for age (*P*<.001), Hukou (*P*<.001), marital status (*P*<.001), education level (*P*<.001), and pension (*P*<.001). Specifically, nearly four-fifths of MOA participants (486/613, 79.3%) were aged 60‐69 years, while less than 50% (294/592, 48.9%) of LOAs were in that age group. In total, 525 (85.6%) of MOAs were rural Hukou while 468 (79.1%) of LOAs were urban Hukou; in addition, there were more than twice as many single LOAs than single MOAs (n=160, 27% vs n=74, 12.1%); over one-fourth of MOAs (n=161, 26.2%) were illiterate, while approximately one-tenth of LOAs (n=69, 11.7%) were illiterate. Finally, 471 (79.6%) LOAs had a pension while only 121 (51.2%) MOAs had one.

### Oral Health, Social Support, and Loneliness of the Participants

Table 2 illustrates the general characteristics of participants’ oral health, life satisfaction, and loneliness, and the difference between MOAs and LOAs for the above variables. The total scores of GOHAI, SSRS, and ULS-6 for MOAs were 54.95 (SD 6.469), 38.89 (SD 6.629), 8.58 (SD 3.032), and 54.40 (SD 7.024), 39.51 (SD 6.856), and 8.00 (SD 2.790) for LOAs. Statistical differences between MOAs and LOAs were found in total ULS-6 score (*t*_1203_=3.442, *P*=.001), SSRS score (objective support*: t*_1203_=4.545, *P*<.001; subjective support*: t*_1203_=−3.608, *P*<.001), and GOHAI score (psychosocial function*: t*_1203_=2.028, *P*=.04). It is noted that there were no statistically significant differences in Q4 (*t*_1203_=1.760, *P*=.08) and Q6 (*t*_1203_=1.265, *P*=.21) of the total score of loneliness between MOAs and LOAs.

**Table 2.** General characteristics of the loneliness, social support, and oral health of MOA^a^ and LOA^b^ participants.

Variables	Total (n=1205), mean (SD)	MOA (n=613), mean (SD)	LOA (n=592), mean (SD)	*t* test (*df*=1203)	*P* value
**Loneliness (ULS-6** ^**c**^ **)**
Total	8.29 (2.929)	8.58 (3.032)	8.00 (2.790)	3.442	.001
Often feel a lack of friends	1.45 (0.757)	1.53 (0.789)	1.36 (0.714)	3.704	<.001
Feel no one can be trusted	1.42 (0.722)	1.47 (0.728)	1.37 (0.713)	2.326	.02
Often feel left out	1.32 (0.587)	1.37 (0.622)	1.28 (0.547)	2.672	.008
Feel separated from others	1.34 (0.654)	1.37 (0.670)	1.30 (0.636)	1.760	.08
Often feel shy	1.31 (0.583)	1.36 (0.636)	1.25 (0.518)	3.365	.001
Surrounded but no one cares	1.46 (0.714)	1.49 (0.720)	1.43 (0.707)	1.265	.21
**Social support (SSRS** ^**d**^ **)**					
Total	39.20 (6.746)	38.89 (6.629)	39.51 (6.856)	−1.612	.11
Objective support	8.22 (2.005)	8.47 (1.636)	7.95 (2.297)	4.545	<.001
Subjective support	23.94 (4.627)	23.47 (4.789)	24.43 (4.404)	−3.608	<.001
Utilization of support	7.04 (2.347)	6.94 (2.257)	7.14 (2.435)	−1.432	.15
**Oral health (GOHAI** ^**e**^ **)**					
Total	54.68 (6.750)	54.95 (6.469)	54.40 (7.024)	1.408	.16
Physical function	17.14 (3.567)	17.35 (3.442)	16.93 (3.683)	2.028	.04
Psychological function	24.05 (2.217)	24.10 (2.061)	23.99 (2.369)	0.832	.41
Pain and discomfort	13.49 (2.162)	13.50 (3.115)	13.48 (2.211)	0.196	.84

a MOA: migrant older adults.

b LOA: local older adults.

c ULS-6: 6-item short-form UCLA Loneliness Scale.

d SSRS: Social Support Rating Scale.

e GOHAI: Geriatric Oral Health Assessment Index.

### Structural Equation Model

#### Measurement Invariance Across Migration Status

Table 3 shows 5 selected models, which revealed related fit statistics of the measurement invariance across migration status and the fitness indexes. The fitness indexes of MOAs and LOAs should be compared to check whether the variable “migration state” was suitable for the group comparison.

In this study, *χ*^*2*^, *df*, *P* value, GFI, AGFI, CFI, and RMSEA were the fitness indexes. As shown in Table 3, the fitness indexes of the MOAs were GFI=0.969, AGFI=0.952, CFI=0.971, and RMSEA=0.045 (M1), while for the LOAs, they were GFI=0.964, AGFI=0.944, CFI=0.940, and RMSEA=0.051 (M2). All fitness indexes showed values over 0.90 and very slight differences between the MOA and LOA groups, implying that we could further compare the differences between the MOA and LOA groups with the other models. Although the RMSEA value of M2 was more than 0.05, these variables were mainly used to calculate the change of RMSEA, not to assess the model’s fitness. Then, the ∆CFI and ∆RMSEA between M3 (unconstrained model), M4 (measurement weights model), and M5 (structural weights model) were used to evaluate the measurement invariance. The M3 did not restrict any coefficient in the model, the M4 assumed the indicator loadings for the corresponding construct of each group are equal, and the M5 constrained the indicator loadings of the corresponding construct and the structural coefficients between the groups.

As seen in Table 3, the ∆CFI between M4 and M3 was 0.002; between M5 and M4, it was 0. All of the ∆CFI values were less than 0.010, indicating that measurement invariance was established between the models of M1, M2, M3, M4, and M5 between the MOA and LOA groups. The ∆RMSEA between M4 and M3 was 0.001, and it was 0 between M5 and M4. All of the ∆RMSEA values were less than 0.015, also indicating that measurement invariance was established between the models of M1, M2, M3, M4, and M5 across the MOA and LOA groups.

**Table 3.** Multigroup model invariance. The variables in 5 models were oral health, social support, and loneliness among MOAs^a^ and LOAs^b^ (n=1205).

Model	Chi-square (*df*)	*P* value	Chi-square*/df*	GFI^c^	AGFI^d^	CFI^e^	RMSEA^f^	∆CFI^g^	∆RMSEA^h^
M1^i^	112.152 (50)	<.001	2.243	0.969	0.952	0.971	0.045	—^j^	—
M2^k^	127.660 (50)	<.001	2.553	0.964	0.944	0.940	0.051	—	—
M3^l^	239.812 (100)	<.001	2.398	0.967	0.948	0.967	0.034	—	—
M4^m^	255.289 (109)	<.001	2.682	0.965	0.950	0.965	0.033	0.002	0.001
M5^n^	258.639 (112)	<.001	2.309	0.964	0.950	0.965	0.033	0.000	0.000

a MOAs: migrant older adults.

b LOAs: local older adults.

c GFI: goodness of fit index.

d AGFI: adjusted goodness of fit index.

e CFI: comparative fitness index.

f RMSEA: root mean square error of approximation.

g ∆CFI: change of CFI.

h ∆RMSEA: change of RMSEA.

i M1: MOA model.

j Not applicable.

k M2: LOA model.

l M3: unconstrained model.

m M4: measurement weights model.

n M5: structural weights model.

#### Model Fitness Indexes

**Figure 1.**
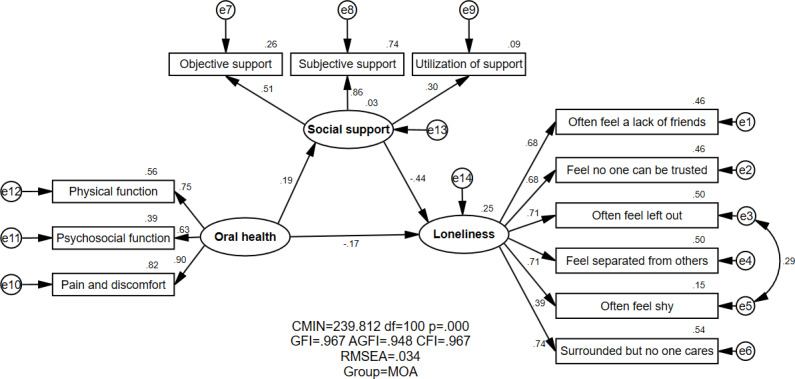
Structural equation modeling analysis of the association between oral health, social support, and loneliness of MOAs (n=613). AGFI: adjusted goodness of fit index; CFI: comparative fitness index; CMIN: chi-square value; e: residual variables; GFI: goodness of fit index; MOA: migrant older adults; RMSEA: root mean square error of approximation. All parameter estimates were statistically significant (*P*<.05).

Figures1  2 show the proposed models for MOAs and LOAs, respectively, which contained 3 variables: oral health, social support, and loneliness. Table 3 demonstrates the model fitness indexes for variable models (M1=MOA, M2=LOA). The MOA and LOA groups both had the same estimated value for model fitness: GFI=0.967, AGFI=0.948, CFI=0.967, and RMSEA=0.034. All fitness indexes implied that the theoretical model perfectly matched the empirical data for both the MOA and LOA groups.

**Figure 2.**
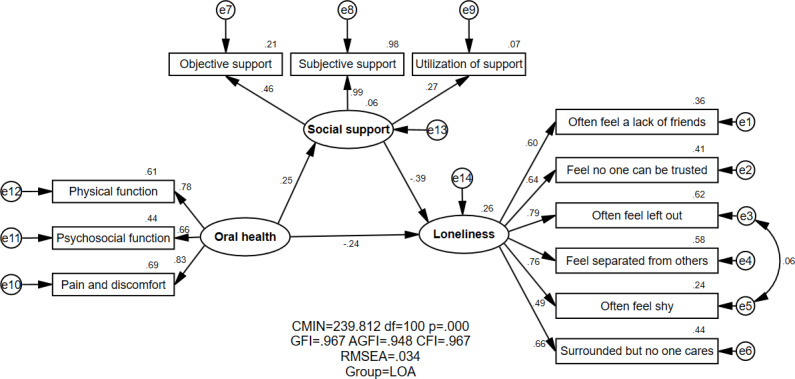
Structural equation modeling analysis of the association between oral health, social support, and loneliness of LOAs (n=592). AGFI: adjusted goodness of fit index; CFI: comparative fitness index; CMIN: chi-square value; e: residual variables; GFI: goodness of fit index; LOA: local older adult; RMSEA: root mean square error of approximation. All parameter estimates were statistically significant (*P*<.05).

### Relationship Between Oral Health, Social Support, and Loneliness Assessed by SEM

#### Association Between Oral Health and Loneliness of Participants

The association between oral health, social support, and loneliness was shown in Figures1  2 and Table 4. A negative and direct association was observed between oral health and loneliness among the MOAs (standardized direct effect=−0.168) and LOAs (standardized direct effect=−0.243). Moreover, oral health could exert a negative effect on loneliness indirectly via social support (standardized indirect effect=−0.083 for MOAs; standardized indirect effect=−0.097 for LOAs). It is noted that oral health was negatively associated with loneliness, which meant that MOAs and LOAs with higher oral health would generally have lower loneliness. A statistically significant relationship between oral health and loneliness was found in both the MOA and LOA groups.

**Table 4.** Standardized effects between oral health, social support, and loneliness among MOAs^a^ and LOAs^b^.

Path	Direct effect		Indirect effect		Total effect		Difference (critical ratio)
	MOA	LOA	MOA	LOA	MOA	LOA	
Oral health → Loneliness	−0.168^c^	−0.243^c^	−0.083^c^	−0.097^c^	−0.251^c^	−0.340^c^	−0.538
Oral health → Social support	0.186^c^	0.247^c^	—^d^	—	0.186^c^	0.247^c^	1.145
Social support → Loneliness	−0.444^c^	−0.392^c^	—	—	−0.444^c^	−0.392^c^	2.741^e^

a MOAs: migrant older adults.

b LOAs: local older adults.

c *P*<.001.

d Not applicable.

e *P*<.01.

#### Association Between Social Support and Loneliness of Participants

As for the relationship between social support and loneliness, a negative and direct effect was demonstrated among both MOAs (standardized direct effect=−0.444) and LOAs (standardized direct effect=−0.392), which meant less social support among both MOAs and LOAs would generally indicate higher loneliness. Concerning the group difference, a significantly negative correlation was slightly stronger in the MOA group than in the LOA group (critical ratio=2.741, *P*<.01).

#### Association Between Oral Health and Social Support of Participants

Oral health had a positive and direct effect on social support for both MOAs and LOAs (standardized direct effect=0.186 for MOAs; standardized direct effect=0.247 for LOAs), indicating that the higher the oral health of MOAs and LOAs, the higher their social support. It was found that a statistically significant relationship existed between oral health and social support among MOAs and LOAs.

## Discussion

### Principal Findings

This study examined the severity of loneliness as well as the association between social support, oral health, and loneliness among older adults in Weifang. The results further showed a statistical difference in loneliness between MOAs and LOAs; the empirical associations between oral health, social support, and loneliness (including the local-migrant difference) were also clarified.

### Loneliness Among MOAs and LOAs

The mean score of loneliness among MOAs (8.58) and LOAs (8.00) was lower than in a previous study conducted among rural empty-nest older adults in China (16.19) [51], indicating a lower level of loneliness among the MOAs and LOAs in this study. Moreover, this study found loneliness in MOAs was higher than in LOAs, which was similar to a study that showed that immigrant groups were lonelier than older adults born in Canada [59]. This may be due to the fact that, because of MOAs’ migration, they need more time to adapt to their new environment while LOA have been in a familiar environment for a long time.

### Association Between Oral Health and Loneliness

A negative association between oral health and loneliness was found among both MOAs and LOAs, which was similar to one existing study, which reported that older people who had a poor oral health status had higher odds of experiencing loneliness [60]. Ma and Chen [27] found that masticatory function, swallowing function, tooth loss, tooth function, and toothache were the influencing factors of loneliness among Chinese older people in the community. Another cross-sectional study in Indonesia showed similar findings, where older adults who had a poor oral status had a higher chance of feeling lonely [61]. Some studies pointed out that poor oral health increases psychological stress in communication among older adults, consequently limiting social interaction with others and causing loneliness [62,63]. However, the results showed that the effect of oral health on loneliness was not statistically different between MOAs and LOAs. This may be due to the fact that the relationship between oral health and loneliness is generalizable across older populations [25]. Another possible reason is that when facing oral health problems, both MOAs and LOAs will adopt different coping styles to decrease their loneliness. Some studies suggested that people with higher socioeconomic status and health literacy tend to proactively utilize medical resources to mitigate the negative impacts of oral problems [64]. In contrast, MOAs had poorer oral health services in their hometowns [65] and have experienced and adapted to more oral problems, which were more common and socially acceptable in their lives [66]. These further result in fewer psychological changes and lower loneliness even though oral problems occurred in the inflow cities.

### Association Between Social Support and Loneliness

A negative relationship between social support and loneliness was found among both MOAs and LOAs, indicating that older adults with higher social support could reduce their loneliness. This was consistent with a previous nationwide cohort study, which also showed that social support decreased the odds of loneliness incidence among older adults in China [67]. The effect of social support on loneliness could be explained through the interaction theory, which views loneliness as a response to a lack of satisfying social networks and attachment partners [68]. Moreover, this study showed that the negative association between social support and loneliness was stronger among the MOAs than the LOAs. The economic development gap between urban and rural areas led to higher medical and education services in urban areas, which further resulted in population mobility mainly from rural to urban areas in China [69,70]. As for the MOAs, their migration may lead to a decline in the quantity and quality of social connections [71], which may further result in higher levels of loneliness among them than in the LOAs.

### Association Between Oral Health and Social Support

The SEM results illustrated that oral health and social support were positively correlated, implying the better the oral health status, the higher the social support. This was consistent with previous findings, which suggested the experience of oral pain was associated with physical discomfort [72] and social barriers [73]. In addition, people with poor oral health may face additional mental health challenges due to problems such as halitosis, creating social anxiety and exacerbating barriers to interaction [74]. The results also noted that no significant difference was found between LOAs and MOAs when examining the impact of oral health on loneliness. This meant that the gradual loss of teeth and changes in facial morphology in older adults as they age, whether migrating or not, could lead to similar social limitations and increased psychological stress, which can affect their social participation and social support.

### Implications

In order to reduce older adults’ loneliness, the following measures should be taken. First, this study found that MOAs had higher loneliness than LOAs. Hence, strengthening social integration and promoting equality may be beneficial for reducing the loneliness of MOAs. Second, the results indicated that loneliness was negatively correlated with oral health; thus, it is important to aid older adults in maintaining their oral health. The government could speed up the process of including oral health services in health insurance reimbursements for older patients and enhance oral health education for them. Third, the findings showed that high social support would also reduce the loneliness of older adults. In particular, for MOAs, their social networks will be affected and they may face challenges such as language barriers and cultural differences after migration, making it more urgent for them to implement effective measures to alleviate their loneliness. Therefore, there is a need to enhance social support through the efforts of family members, communities, and the government. For instance, the government could implement policies to support young people whose families include older adults since older adults may receive financial and emotional support from their children. It is necessary to promote the equalization of social security and welfare benefits for older adults who migrate with their families and further enhance their belonging in the inflow city.

### Limitations

Some limitations existed in this study and should be addressed in future research. First, this study used cross-sectional data, therefore causal relationships could not be made and a longitudinal design is needed for follow-up research. Second, oral health status was assessed by a self-report scale with a lack of clinical evidence. In the future, clinical oral health examinations could be used to evaluate the oral health of older adults. Third, in addition to social support, there may be other variables that exert an indirect effect on loneliness among older adults and the variables used in this study may also be influenced by other confounding factors; therefore, more research is needed to verify their association.

### Conclusion

Loneliness levels were fairly low among older adults in Weifang, China, while MOAs showed higher loneliness than LOAs. Oral health had both direct and indirect negative effects on loneliness among MOAs and LOAs, with no significant path differences between MOAs and LOAs. Social support was found to be negatively associated with loneliness for both MOAs and LOAs, while the association was stronger among MOAs than LOAs. Oral health exerted a significantly positive effect on social support for both MOAs and LOAs, while no significant difference existed between LOAs and MOAs.

## Supplementary material

10.2196/66061 Multimedia Appendix 1 A flow diagram of the sample selection process.

## Abbreviations

AGFI: adjusted goodness of fit Index
CFI: comparative fitness index
CMIN: chi-square value
GFI: goodness of fit index
GOHAI: Geriatric Oral Health Assessment Index
LOA: local older adults
MOA: migrant older adults
RMSEA: root mean square error of approximation
SSRS: Social Support Rating Scale
ULS-6: 6-item short-form UCLA Loneliness Scale
