# Supplementary figures and images for: Migrant-Local Differences in the Relationship Between Oral Health, Social Support, and Loneliness Among Older Adults in Weifang, China: Cross-Sectional Study
Source: JMIR Public Health Surveill. 2025 Mar 13;11:e66061. doi: 10.2196/66061 (PMC11925387; doi:10.2196/66061)

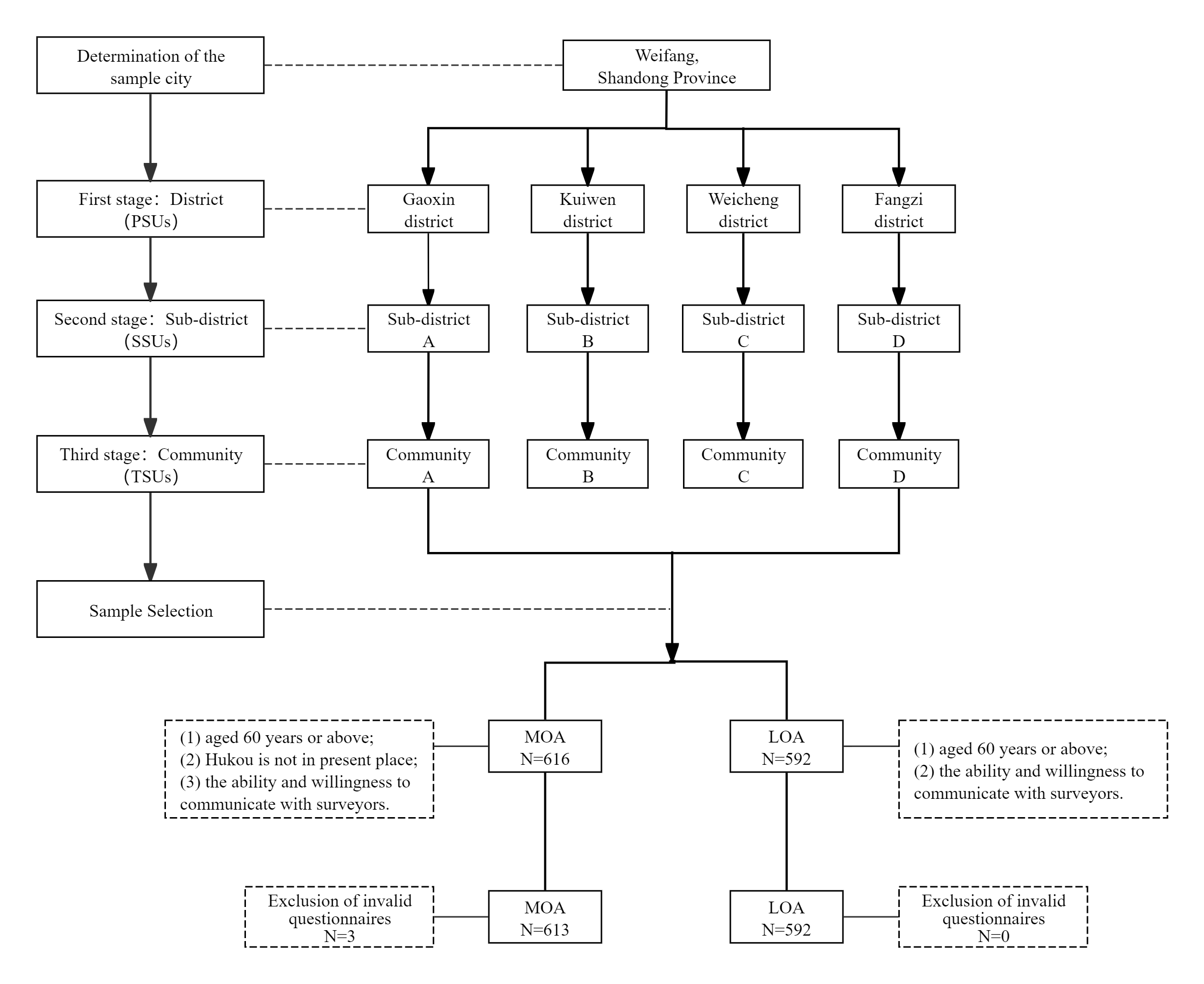

Supplement: Multimedia Appendix 1 [file publichealth-v11-e66061-s001.png]
